# A Comparative Analysis of Alkaline Phosphatase Levels in Gingival Crevicular Fluid of Patients Undergoing Growth Modulation Therapy With Twin Block, Forsus Fatigue Resistant, and Clear Block Appliances Compared to Normal Individuals: An In Vivo Study

**DOI:** 10.7759/cureus.63374

**Published:** 2024-06-28

**Authors:** Ayush Sundrani, Ranjit Kamble, Dhwani Suchak, Japneet Kaiser, Nishu Agarwal, Nandalal Toshniwal

**Affiliations:** 1 Orthodontics and Dentofacial Orthopaedics, Sharad Pawar Dental College, Datta Meghe Institute of Higher Education and Research, Wardha, IND; 2 Orthodontics and Dentofacial Orthopaedics, Smile Care Centre, Raipur, IND; 3 Orthodontics and Dentofacial Orthopaedics, Rural Dental College, Pravara Institute of Medical Sciences (Deemed to be University), Loni, IND

**Keywords:** alkaline phosphatase (alp), orthodontic appliances, bone turnover, growth modulation therapy, gingival crevicular fluid (gcf)

## Abstract

Background

In the contemporary era, where science and technology know no boundaries, this in vivo study explores the impact of growth modulation therapy using Twin Block, Forsus Fatigue Resistant, and Clear Block appliances on alkaline phosphatase (ALP) levels in gingival crevicular fluid (GCF). Bone physiology involves modeling and remodeling, with orthodontics applying forces to teeth, influencing tissue reactivity and bone modeling. ALP, a marker of osteoblast function, plays a crucial role in bone growth. GCF reflects immunological and inflammatory responses during orthodontic force application, making it a valuable medium for studying ongoing metabolic processes related to bone turnover.

Aim

The study aims to comparatively analyze ALP levels in GCF during growth modulation therapy, assessing the efficacy of Twin Block, Forsus Fatigue Resistant, and Clear Block appliances. The research involves 30 experimental samples divided into three study groups and a control group. The samples are collected at various time intervals, and ALP levels are analyzed using a spectrophotometer. Statistical analysis includes paired and unpaired t-tests, one-way analysis of variance (ANOVA), and multiple comparisons.

Results

Results demonstrate a significant increase in ALP levels during the growth modulation therapy, indicating a positive correlation with bone remodeling. Twin Block appears to be the most effective appliance, exhibiting higher ALP activity compared to Clear Block and Forsus groups.

Conclusion

In conclusion, this study provides valuable insights into the biochemical responses during growth modulation therapy, emphasizing the potential of GCF analysis in understanding orthodontic treatment effects.

## Introduction

In the contemporary era, science and technology face no boundaries [1]. In the realm of bone physiology, modeling is the process that shapes structures by utilizing the raw material of bone formation, influenced by developmental programs and mechanical loading. Remodeling serves as the mechanism that regulates skeletal turnover and repair throughout one's life [2]. Orthodontics concentrates on applying forces to teeth and associated tissues. The frequency, amount, and duration of orthodontic therapy exert varying impacts on the reactivity of adjacent tissues and bone modeling [3]. Within bone modeling, the interaction of bone growth and resorption during tooth movement leads to the release of diverse biochemical or cellular mediators, potentially serving as identifiable biomarkers [3]. Bone biomarkers like alkaline phosphatase (ALP) are often associated with bone development. ALP plays a crucial role by hydrolyzing pyrophosphate groups to inorganic phosphate groups, promoting osteoblastic mineral formation [4]. Quantities of bone-specific alkaline phosphatase (BALP) produced by osteoblasts can be quantified to assess bone production and turnover, offering a means of gauging treatment response in clinical settings [5].

Gingival crevicular fluid (GCF) has been found to contain indicators of bone remodeling and breakdown. Therefore, GCF has been shown to reflect immunological and inflammatory responses during both periodontitis and orthodontic force application. Forces applied during orthodontic treatment disrupt the periodontal ligament's extracellular matrix, influencing the cellular structure and cytoskeletal topologies. These alterations may impact the flow rate and composition of GCF [6]. GCF is considered to be a transudate of interstitial tissues created by an osmotic gradient and released into the crevicular sulcus at an approximate flow rate of 3 µL/h. However, during periodontal inflammation, GCF synthesis primarily becomes exudative, resulting in an increased flow rate and volume, reaching up to 44 µL/h [7]. Examining GCF samples proves to be a valuable method for investigating the ongoing metabolic processes related to bone turnover during orthodontic tooth movement. If it were possible to biologically assess and predict the consequences of orthodontic forces, appliance maintenance could be tailored to individual tissue reactions, thereby enhancing the efficacy of the orthodontic procedure. Additionally, addressing retention issues could involve monitoring the bone turnover rate around the teeth [8]. The collection of GCF samples is a straightforward, uncomplicated, and reproducible technique with minimal risk to the patient. Consequently, the present study aims to analyze ALP levels in the GCF of patients undergoing growth modulation therapy with various growth-modulating devices and to explore any correlations during the course of growth modulation therapy at different time intervals.

Aim

The aim was to comparatively analyze the ALP levels in the GCF of patients undergoing growth modulation therapy with Twin Block, Forsus Fatigue Resistant, and Clear Block appliances, as compared to normal individuals.

Objectives

The objectives of the study were as follows: (1) to assess the ALP levels in GCF during growth modulation therapy using Twin Block appliance therapy, Clear Block appliance therapy, and Forsus Fatigue Resistant appliance therapy, and (2) to compare the ALP levels in GCF at varied periods during growth modulation therapy using Twin Block appliance therapy, Clear Block appliance therapy, and Forsus Fatigue Resistant appliance therapy.

The study was conducted in the Department of Orthodontics and Dentofacial Orthopaedics, Sharad Pawar Dental College, Sawangi (Meghe), in collaboration with Central Clinical Research Laboratory, Jawaharlal Nehru Medical College, Sawangi (Meghe), and Sarasvati Pathology Lab, Nagpur, India. The study was approved by the Institutional Ethics Committee of Datta Meghe Institute of Higher Education and Research issued the approval DMIMS (DU)/IEC/2015-16/1522, dated 5/10/2015. Informed consent was obtained from the patient's parents.

## Materials and methods

Sample selection

Thirty experimental samples with Angle's Class II malocclusion and functional retrusion of the mandible were selected from the Outpatient Department (OPD). These were further divided into three study groups of 10 each for Twin Block, Clear Block, and Forsus Fatigue Resistant appliances, with a control group comprising 15 patients. The patients with Angle’s Class II malocclusion with functional retrusion of the mandible, patients aged 10 to 14 years (both males and females), positive visual treatment objective (VTO) on clinical evaluation, and patients with no history of orthodontic treatment, trauma during childhood (birth injury), temporomandibular joint (TMJ) dysfunction or infection during childhood, and cases having the following cephalometric parameters (angle SNA = 82° ± 2°, angle SNB < 76°, angle ANB > 5°) were selected for the study. Patients with generalized systemic disorders, patients with a history of liver or bone diseases, healing fractures, or thyroid disorders, which may show increased levels of ALP in the body, patients affected with craniofacial syndromes, and patients with poor oral hygiene and periodontal condition, were excluded from the study.

Complete case history and clinical examination (intraoral and extraoral examinations) of the selected cases were done. The lateral cephalograms of the selected cases were traced, digitized, and analyzed with computer software to assess the skeletal malocclusion. The cases selected had the following cephalometric parameters: angle SNA = 82° ± 2°, angle SNB < 76°, angle ANB > 5°.

**Figure 1:**
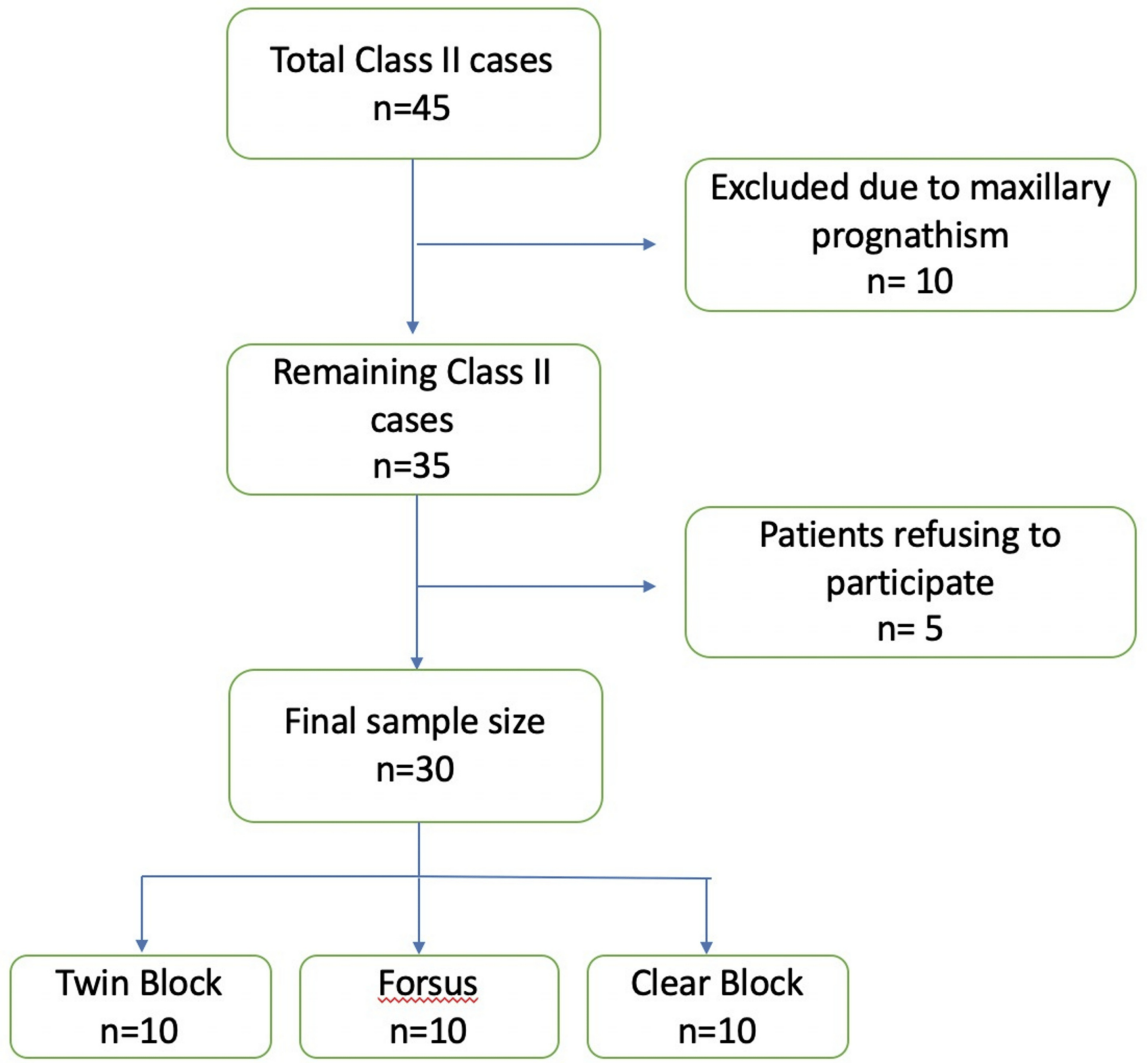
Sample size selection criteria

**Figure 2:**
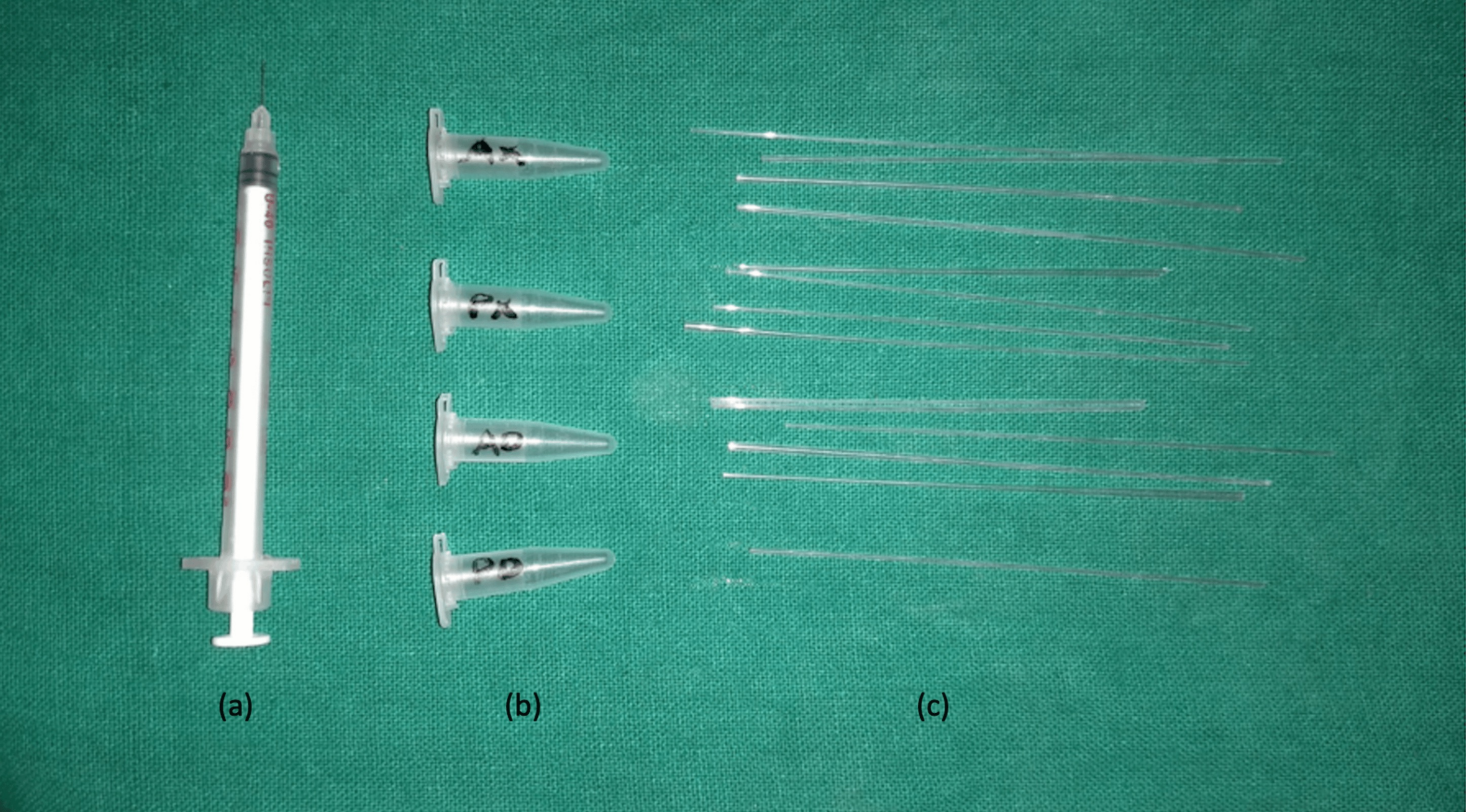
(a) Insulin syringe; (b) Eppendorf tubes for dispensing the GCF out of the tubes; (c) Capillary tubes for collecting the GCF GCF: Gingival crevicular fluid

**Figure 3:**
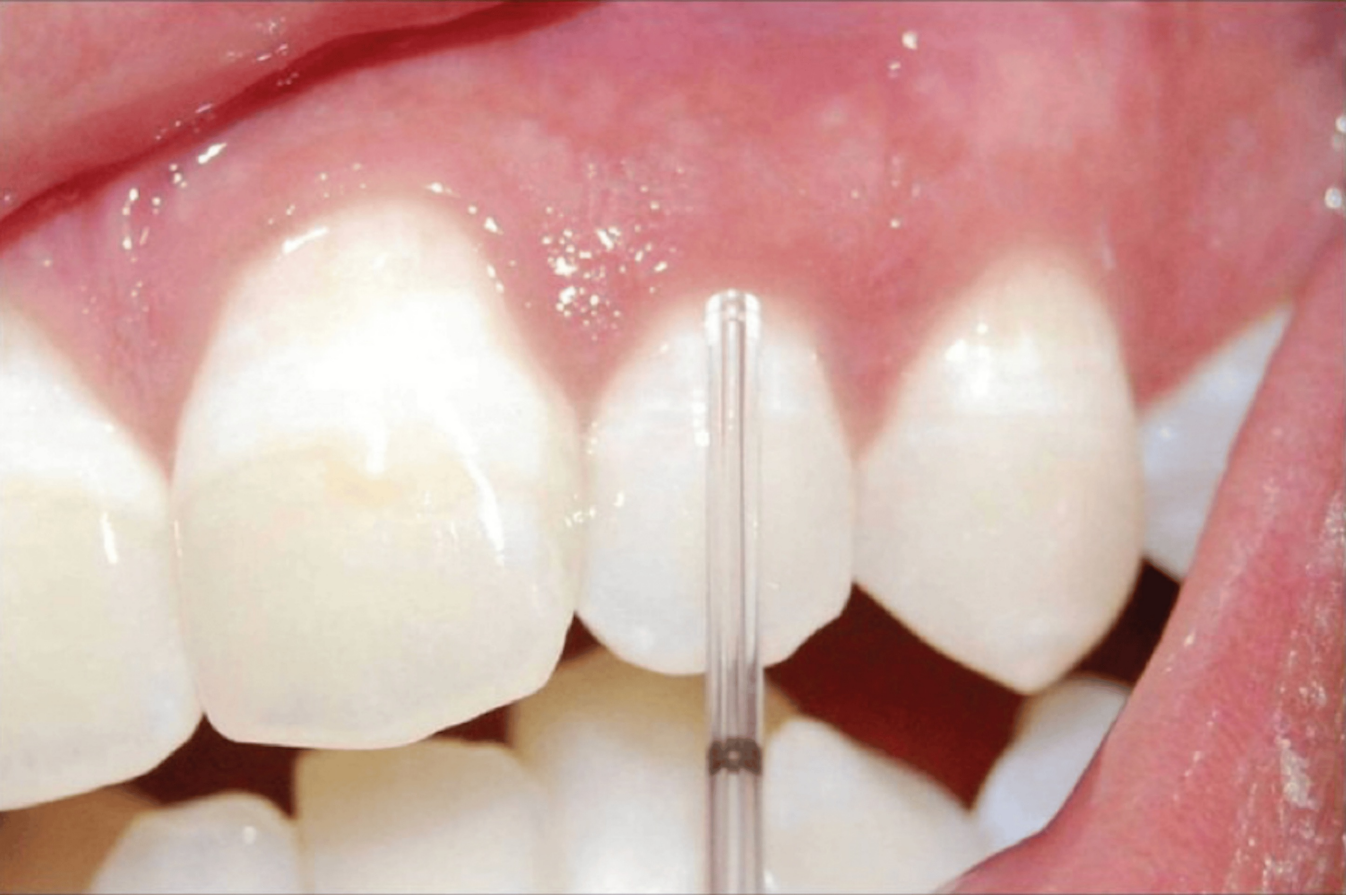
Collecting GCF by capillary tubes GCF: Gingival crevicular fluid

A total of 30 Skeletal Class II Division 1 cases were selected, and myofunctional therapy was given (Figure 1). The lateral cephalograms were taken by a Planmeca Proline CC (Planmeca, Helsinki, Finland) machine. The canines and first molars, of both the maxilla and mandible, were selected as sampling sites. Supragingival plaque was removed, and the region was flushed with water and gently air-dried. The sites were isolated using cotton rolls and gently dried for five seconds. From each test site, GCF was collected using capillary tubes (Figure 2). The tubes were inserted into the gingival sulcus and held for two minutes. The collected GCF was then transferred to Eppendorf tubes using an insulin syringe (Figure 3).

**Figure 4:**
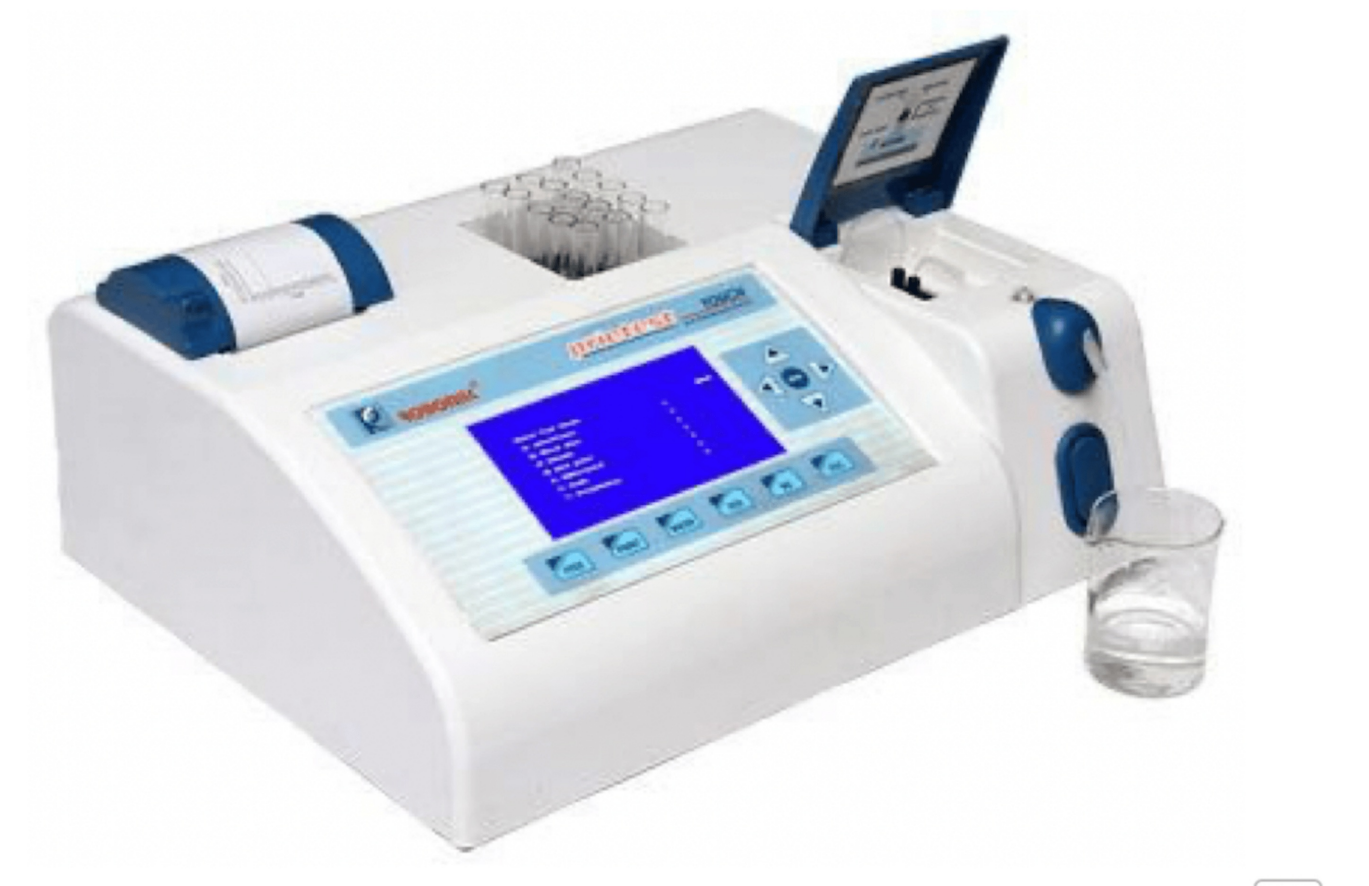
Spectrophotometer

**Figure 5:**
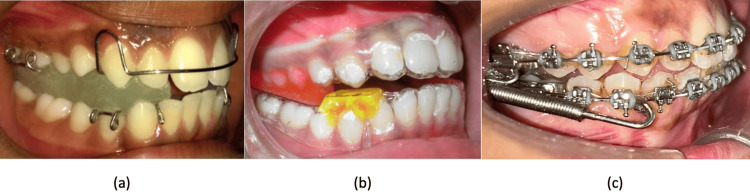
Clinical photographs of patients with different advancement appliances (a) Patients with Twin Block; (b) Patients with Clear Block; (c) Patients with Forsus Fatigue Resistant device

These samples were collected at the following time intervals: T0 - Pre-treatment, T1 - after one week of appliance delivery, T2 - after one month, T3 - after three months, and T4 - after six months. The GCF was stored at -20ºC until biochemically assayed and analyzed by a spectrophotometer (biochemical analyzer) (Figure 4). The facilities at the central clinical research lab of Jawaharlal Nehru Medical College were utilized for this biochemical analysis. Estimation of ALP levels was done using the ALP activity test according to IFCC recommendation (Figure 5) [9].

Method

This approach involves employing 4-nitrophenyl phosphate as the substrate. When operating under ideal conditions, the ALP enzyme in the sample triggers the ensuing reaction.

At the reaction's pH, 4-nitrophenyl phosphate displays a vibrant yellow hue. The reagent is equipped with a metal ion buffer system to uphold optimal concentrations of zinc and magnesium. Additionally, the metal ion buffer can bind to other potentially inhibitory ions that might be present. The progression of the reaction is tracked by gauging the rate of absorbance increase at 405 or 415 nm at 37°C, directly corresponding to the activity of ALP in the saliva.

A statistical examination was conducted to compare the levels of ALP in the GCF among individuals undergoing growth modulation therapy with Twin Block, Clear Block, and Forsus Fatigue Resistant appliances, in comparison to those in normal individuals. The analysis aimed to identify any significant differences in these values. The software used in the analysis was SPSS Statistics for Windows, Version 17 (Released 2008; SPSS Inc., Chicago, USA), and GraphPad Prism 5.0 version (GraphPad Software, San Diego, USA), and p < 0.05 is considered as the level of significance. The statistical tests used for the analysis of the results were: 1) Student's paired t-test, 2) Student's unpaired t-test, 3) One-way ANOVA, and 4) Tukey's multiple comparison test.

## Results

Table 1 shows the comparison of ALP activity with the Forsus Fatigue Resistant appliance in the study and control groups. Table 2 shows the ALP activity at different time intervals in the Forsus Fatigue Resistant appliance group compared to T0. The ALP activity at T1, T2, T3, and T4, when compared to T0, was statistically significant at all four collection sites.

**Table 1:** Comparison of alkaline phosphatase activity with Forsus Fatigue Resistant appliance in study and control groups FFR-S: Forsus Fatigue Resistant-study group; FFR-C: Forsus Fatigue Resistant-control group; AX: Anterior maxilla; PX: Posterior maxilla; AD: Anterior mandible; PD: Posterior mandible; T0: Pre-treatment; T1: After 1 week of appliance delivery; T2: After 1 month, T3: After 3 months; T4: After 6 months p < 0.05 = significant; p > 0.05 = non-significant

	FFR-S	FFR-C	t-value	p-value
T0				
AX	101.70±3.52	99.40±2.40	1.30	0.21
PX	112.40±4.71	105.80±1.92	2.96	0.011
AD	113.30±3.62	104.60±3.04	4.59	0.0001
PD	111.70±4.44	103±2.23	4.06	0.001
T1				
AX	114.40±4.32	110.80±1.64	1.77	0.10
PX	134.10±3.44	119.60±1.14	9.01	0.0001
AD	132.50±3.65	118±1.58	8.35	0.0001
PD	122.90±2.99	118.6±2.30	2.80	0.015
T2				
AX	132.40±3.37	107.80±1.48	15.35	0.0001
PX	151.50±3.89	114.80±2.49	19.02	0.0001
AD	154.80±2.78	114.60±2.70	26.62	0.0001
PD	134.40±2.75	117.20±3.11	10.93	0.0001
T3				
AX	154.10±2.88	104.80±1.92	34.26	0.0001
PX	172.20±3.22	112.20±3.11	34.32	0.0001
AD	171.60±2.06	113.20±4.65	34.35	0.0001
PD	149.10±2.46	114.20±3.27	23.24	0.0001
T4				
AX	140.30±2.31	101.80±3.76	24.75	0.0001
PX	164.20±2.48	108.40±3.50	35.88	0.0001
AD	163.80±1.87	110.60±6.84	23.67	0.0001
PD	141.20±1.54	110.20±7.39	13.16	0.0001

**Table 2:** Comparison of alkaline phosphatase activity with Forsus Fatigue Resistant appliance in the study group at different time intervals with T0 AX: Anterior maxilla; PX: Posterior maxilla; AD: Anterior mandible; PD: Posterior mandible; T0: Pre-treatment; T1: After 1 week of appliance delivery; T2: After 1 month, T3: After 3 months; T4: After 6 months p < 0.05 = significant; p > 0.05 = non-significant

Sites		Mean	N	Std. deviation	Std. error mean	t-value	p-value
AX	T0	101.7	10	3.52	1.11	-	-
	T1	114.4	10	4.32	1.36	12.83	0.0001
	T2	132.4	10	3.37	1.06	28.27	0.0001
	T3	154.1	10	2.88	0.91	28.21	0.0001
	T4	140.3	10	2.31	0.73	32.06	0.0001
PX	T0	112.4	10	4.71	1.49	-	-
	T1	134.1	10	3.44	1.08	11.13	0.0001
	T2	151.5	10	3.89	1.23	18.43	0.0001
	T3	172.2	10	3.22	1.01	29.27	0.0001
	T4	164.2	10	2.48	0.78	24.96	0.0001
AD	T0	113.3	10	3.62	1.14	-	-
	T1	132.5	10	3.65	1.15	9.52	0.0001
	T2	154.8	10	2.78	0.87	27.53	0.0001
	T3	171.6	10	2.06	0.65	35.69	0.0001
	T4	163.8	10	1.87	0.59	34	0.0001
PD	T0	111.7	10	4.44	1.4	-	-
	T1	122.9	10	2.99	0.94	9.19	0.0001
	T2	134.4	10	2.75	0.87	18.73	0.0001
	T3	149.1	10	2.46	0.78	24.34	0.0001
	T4	141.2	10	1.54	0.48	18.04	0.0001

Table 3 shows a comparison of ALP activity with the Twin Block appliance in the study and control groups. Table 4 shows the ALP activity at different time intervals in the Twin Block appliance group compared to T0. The ALP activity at T1, T2, T3, and T4 when compared to T0 was statistically significant at all four collection sites.

**Table 3:** Comparison of alkaline phosphatase activity with Twin Block appliance in the study and control groups TB-S: Twin Block-study group; TB-C: Twin Block-control group; AX: Anterior maxilla; PX: Posterior maxilla; AD: Anterior mandible; PD: Posterior mandible; T0: Pre-treatment; T1: After 1 week of appliance delivery; T2: After 1 month, T3: After 3 months; T4: After 6 months p < 0.05 = significant; p > 0.05 = non-significant

	TB-S	TB-C	t-value	p-value
T0				
AX	104.40±5.56	100.20±2.77	1.57	0.14
PX	109.90±2.46	105.20±2.48	3.46	0.004
AD	113.90±2.33	104.80±2.38	7.07	0.0001
PD	106.20±2.85	103.80±2.38	1.60	0.13
T1				
AX	118±5.27	109.80±1.48	3.35	0.005
PX	123.80±1.93	120.20±1.78	3.47	0.004
AD	133.30±3.12	117.40±1.94	10.29	0.0001
PD	124.60±2.22	117.40±0.89	6.87	0.0001
T2				
AX	135.30±2.98	108.80±1.09	18.93	0.0001
PX	149.40±2.75	115.20±2.38	23.57	0.0001
AD	158.30±3.16	115.20±2.16	27.18	0.0001
PD	145.60±3.30	115.40±2.70	17.59	0.0001
T3				
AX	159.10±1.79	105.40±2.07	52.06	0.0001
PX	172.90±2.88	111.60±2.70	39.55	0.0001
AD	179.20±2.69	113±3.80	39.19	0.0001
PD	158.80±2.57	113.60±3.36	29.06	0.0001
T4				
AX	147.20±1.47	102.80±3.76	33.43	0.0001
PX	163.90±2.51	107.80±3.27	36.98	0.0001
AD	167.80±1.54	111±5.65	30.57	0.0001
PD	145±2.78	110.20±7.36	13.52	0.0001

**Table 4:** Comparison of alkaline phosphatase activity with Twin Block appliance in the study group at different time intervals with T0 AX: Anterior maxilla; PX: Posterior maxilla; AD: Anterior mandible; PD: Posterior mandible; T0: Pre-treatment; T1: After 1 week of appliance delivery; T2: After 1 month, T3: After 3 months; T4: After 6 months p < 0.05 = significant; p > 0.05 = non-significant

Sites		Mean	N	Std. deviation	Std. error mean	t-value	p-value
AX	T0	104.4	10	5.56	1.75	-	-
	T1	118	10	5.27	1.66	27.26	0.0001
	T2	135.3	10	2.98	0.94	15.69	0.0001
	T3	159.1	10	1.79	0.56	29.75	0.0001
	T4	147.2	10	1.47	0.46	27.03	0.0001
PX	T0	109.9	10	2.46	0.78	-	-
	T1	123.8	10	1.93	0.61	15.44	0.0001
	T2	149.4	10	2.75	0.87	31.84	0.0001
	T3	172.9	10	2.88	0.91	43.13	0.0001
	T4	163.9	10	2.51	0.79	41.01	0.0001
AD	T0	113.9	10	2.33	0.73	-	-
	T1	133.3	10	3.12	0.98	32.33	0.0001
	T2	158.3	10	3.16	1	38.71	0.0001
	T3	179.2	10	2.69	0.85	56.52	0.0001
	T4	167.8	10	1.54	0.48	50.4	0.0001
PD	T0	106.2	10	2.85	0.9	-	-
	T1	124.6	10	2.22	0.7	20.51	0.0001
	T2	145.6	10	3.306	1.04	24.51	0.0001
	T3	158.8	10	2.57	0.81	45.86	0.0001
	T4	145	10	2.78	0.88	46.89	0.0001

Table 5 shows a comparison of ALP activity with the Clear Block appliance in the study and control groups. Table 6 shows the ALP activity at different time intervals in the Clear Block appliance group compared to T0. The ALP activity at T1, T2, T3, and T4 when compared to T0 was statistically significant at all four collection sites. 

**Table 5:** Comparison of alkaline phosphatase activity with Clear Block appliance in study and control groups CB-S: Clear Block-study group; CB-C: Clear Block-control group; AX: Anterior maxilla; PX: Posterior maxilla; AD: Anterior mandible; PD: Posterior mandible; T0: Pre-treatment; T1: After 1 week of appliance delivery; T2: After 1 month, T3: After 3 months; T4: After 6 months p < 0.05 = significant; p > 0.05 = non-significant

	CB-S	CB-C	t-value	p-value
T0				
AX	104.80±4.75	101.20±2.58	1.56	0.14
PX	113.10±2.60	106.40±1.81	5.12	0.0001
AD	114.50±3.24	104.20±2.58	6.15	0.0001
PD	110.70±5.37	103.40±2.40	2.85	0.014
T1				
AX	118±4.44	111±3.16	3.12	0.008
PX	132.20±5.02	119.80±1.92	5.24	0.0001
AD	132.80±3.70	117.80±2.16	8.27	0.0001
PD	124.30±2.75	118±1.58	4.69	0.0001
T2				
AX	132.60±2.83	108.20±0.83	18.52	0.0001
PX	150.60±4.14	115±2.23	17.74	0.0001
AD	156.60±2.98	114.60±3.36	24.67	0.0001
PD	138±5.57	116.60±2.70	8.01	0.0001
T3				
AX	155.40±4	104.80±1.92	26.39	0.0001
PX	172.70±3.23	112.40±2.07	37.62	0.0001
AD	173.90±4.88	113.40±3.20	24.88	0.0001
PD	152.20±5.43	114.80±3.11	14.11	0.0001
T4				
AX	141.90±4.53	103±4.06	16.16	0.0001
PX	164.40±2.36	108.80±3.96	34.40	0.0001
AD	164.90±2.84	112±6.67	21.98	0.0001
PD	142.40±2.31	110.40±8.59	11.36	0.0001

**Table 6:** Comparison of alkaline phosphatase activity with Clear Block appliance in the study group at different time intervals with T0 AX: Anterior maxilla; PX: Posterior maxilla; AD: Anterior mandible; PD: Posterior mandible; T0: Pre-treatment; T1: After 1 week of appliance delivery; T2: After 1 month, T3: After 3 months; T4: After 6 months p < 0.05 = significant; p > 0.05 = non-significant

Sites		Mean	N	Std. deviation	Std. error mean	t-value	p-value
AX	T0	104.8	10	4.75	1.5	-	-
	T1	118	10	4.44	1.4	13.86	0.0001
	T2	132.6	10	2.83	0.89	20.74	0.0001
	T3	155.4	10	4	1.26	43.38	0.0001
	T4	141.9	10	4.53	1.43	59.57	0.0001
PX	T0	113.1	10	2.6	0.82	-	-
	T1	132.2	10	5.02	1.59	11.43	0.0001
	T2	150.6	10	4.14	1.3	21.47	0.0001
	T3	172.7	10	3.23	1.02	42.57	0.0001
	T4	164.4	10	2.36	0.74	41.71	0.0001
AD	T0	114.5	10	3.24	1.02	-	-
	T1	132.8	10	3.7	1.17	9.44	0.0001
	T2	156.6	10	2.98	0.94	26.17	0.0001
	T3	173.9	10	4.88	1.54	32.73	0.0001
	T4	164.9	10	2.84	0.9	34.83	0.0001
PD	T0	110.7	10	5.37	1.7	-	-
	T1	124.3	10	2.75	0.86	7.07	0.0001
	T2	138	10	5.57	1.76	9.13	0.0001
	T3	152.2	10	5.43	1.71	13.5	0.0001
	T4	142.4	10	2.31	0.73	14.2	0.0001

## Discussion

Mechanical strain in orthodontics seems to induce biochemical and structural responses in various cell types, both in living organisms and in laboratory settings. The early phase of orthodontic tooth movement is marked by an initial inflammatory reaction, involving periodontal vasodilation and the migration of leukocytes out of capillaries in the periodontal ligament [10]. ALP is considered a crucial marker of osteoblast function essential for bone growth. It facilitates the hydrolysis of pyrophosphate, generating inorganic phosphate that plays a role in mineralization preparation [11,12].

The GCF contains a complicated mixture of chemicals obtained from serum, host inflammatory cells, periodontal cells, and oral microorganisms. Uematsu et al. [13] discovered that orthodontic therapy significantly increased the levels of various inflammatory mediators in GCF, including interleukin 1β, interleukin 6, tumor necrosis factor-α, epidermal growth factor, and β2 microglobulin. Grieve et al. [14] reported comparable findings for prostaglandin E and interleukin 1β.

Tables 1-2 show the ALP levels in GCF collected from four different sites, i.e., anterior maxilla (AX), posterior maxilla (PX), anterior mandible (AD), and posterior mandible (PD), at five-time intervals in the Forsus Fatigue Resistant appliance group and comparison with the control group. The comparison of ALP levels in the AX collection site of the experimental and control groups at times T0 and T1 was statistically insignificant, while at T2, T3, and T4, it was statistically significant. The comparison of PX, AD, and PD collection sites in the experimental and control groups was found to be statistically significant at all five-time intervals. The ALP activity at T1, T2, T3, and T4, when compared to T0, was statistically significant at all four collection sites.

These results were found to be in accordance with the study conducted by Perinetti et al. [15], in which they used a longitudinal design to investigate ALP activity in GCF. The maxillary first molars were used as the test teeth (TT). One of the TT molars, the distalized molar (DM), was to be treated for distal movement, whereas its contralateral molar (CM) was not moved distally. The DM antagonist first molar (AM) was used as a control. At baseline and at one hour, in both distal and mesial sites, ALP activity was similar among the three groups without significant differences. In mesial sites, pair-wise comparisons showed an enzymatic activity significantly greater in the DMs than in the CMs and AMs from days 7 to 28. In the same sites, GCF ALP activity from the CMs was significantly greater than in the AMs from 7 to 21 days. In distal sites, significant differences between the DMs and CMs were seen only on days 14 and 21, whereas the differences between the DMs and AMs were significant from 7 to 28 days. In distal sites, statistically significant differences between the CMs and AMs were seen from day 7 to the end of the experiment.

Wahab et al. [16] encountered conflicting outcomes in their investigation of the influence of different orthodontic forces (100 or 150 gm) on specific ALP activities in GCF and their correlation with the rate of canine movement over a five-week retraction period. In the 150 gm group, no noteworthy differences were found in baseline ALP activity between the experimental and control locations (p > 0.05). Notably, the mesial sites of the experimental canines exhibited peak ALP activity during week 1 under 150 gm force, reaching three times higher levels (p < 0.05) compared to ALP activity in control teeth. The ALP activities of experimental canines during weeks 1 and 2 were significantly elevated compared to baseline. The baseline ALP activity with 100 gm force did not exhibit significant differences (p > 0.05) from control teeth at both locations. ALP activity at the mesial sites reached its peak in week 2 compared to baseline, with the peak enzyme activity at the experimental site being 2.5 times higher than the baseline activity, although these differences were not statistically significant (p > 0.05). ALP activity decreased in week 3 before stabilizing two weeks later. No significant differences (p > 0.05) in enzyme activity were observed between experimental and control teeth at the distal sites.

Tables 3-4 show the ALP levels in GCF collected from four different sites, i.e., AX, PX, AD, and PD, at five-time intervals in the Twin Block appliance group and in comparison with the control group. The comparison of ALP levels in the AX and PD collection sites of the experimental and control group at time T0 was statistically insignificant, while at T1, T2, T3, and T4, it was statistically significant. The comparison of PX and AD collection sites in the experimental and control groups was found to be statistically significant at all five-time intervals. The ALP activity at T1, T2, T3, and T4, when compared to T0, was statistically significant at all four collection sites.

Alfaqeeh and Anil [17] also reported similar results in their longitudinal study in which they assessed the changes in the GCF volume and the levels of ALP during the early phases of tooth movement. It was found to be statistically significant on the 14th day with a peak level of 56.75 ± 11.50 IU/L. On the control side, there was only minimal variation from 0 hours to 21 days in all 20 subjects studied, and the values were not statistically significant.

Batra et al. [18] unearthed findings that opposed the present study during their examination of the alteration in ALP activity throughout 21 days of orthodontic tooth movement. The association between enzyme activity on day 0 and day 1 did not display statistical significance (p = 0.9071) in comparison to the control location (p < 0.05). By the 21st day, the enzyme activity at the experimental site declined to 1.3 times that of the control site (p < 0.05).

Tables 5-6 show the ALP levels in GCF collected from four different sites i.e., AX, PX, AD, and PD, at five-time intervals in the Clear Block appliance group and compare them with the control group. The comparison of ALP levels in the AX collection site of the experimental and control groups at time T0 was statistically insignificant, while at T1, T2, T3, and T4, it was statistically significant. The comparison of PX, AD, and PD collection sites in the experimental and control groups was found to be statistically significant at all five-time intervals. The ALP activity at T1, T2, T3, and T4, when compared to T0, was statistically significant at all four collection sites. 

Abdullah et al. [19] identified comparable outcomes in their longitudinal study on the activity of ALP in GCF during the leveling and alignment phase. Initially, at baseline (week 0), when forces were not applied, both the mesial and distal regions of the experimental teeth exhibited comparable levels of ALP activity. ALP levels in the mesial and distal sites increased to 0.12 U and 0.14 U, respectively, after one week of force application. By the second week, the ALP amount at the mesial location had significantly increased, while the distal sites remained unchanged from the week before.

The results of the present study differed from those of Isik et al. [8], who examined four bone markers (Dpd, cross-linked NTx, osteocalcin, and BALP) in GCF during intrusive tooth movement, hypothesizing bone resorption. GCF samples were collected before appliance fitting and at 1, 24, and 168 hours post-activation. Osteocalcin and BALP values generally decreased after activation, with a slight, non-significant rise on the seventh day.

In all the three appliance groups a gradual increase in the ALP levels was seen from time T0 to T3 and a slight drop in the ALP levels was seen at time T4. These results are suggestive of a positive correlation between the increasing ALP levels and bone remodeling occurring in the maxilla and mandible during growth modulation therapy.

The following limitations can be considered for the study: (a) The study was conducted for six months. Long-term evaluation of changes in ALP levels needs to be studied for a better understanding of its role in the acceleration of growth induced by functional appliances, and (b) the increase in GCF levels of ALP that we found cannot be directly correlated to local production of ALP in the condylar region due to the anatomic constraints and limits of our study, as it was not possible to obtain cartilage samples from humans.

A further study for long-term evaluation needs to be carried out to assess the alterations in ALP levels and its role in mandibular growth during functional appliance therapy, particularly after six months or more, when endochondral bone formation occurs.

## Conclusions

ALP levels were significantly higher at all four other time intervals compared to pre-treatment levels. Peak ALP activity was observed in PD at T0, PX at T1, AD, and PD at T2. At T3 and T4, peak activity was observed in AX, AD, and PD. Pre-treatment showed peak ALP activity in the posterior mandible region, higher in the Forsus Fatigue Resistant group compared to the Twin Block group. At one week (T1), increased ALP activity was seen in the Forsus Fatigue Resistant group, followed by the Clear Block and Twin Block groups. After one month (T2), increased ALP activity was observed in the Twin Block group, followed by the Clear Block and Forsus groups. In the third month (T3) and sixth month (T4), increased ALP activity was observed in the Twin Block group, followed by the Clear Block and Forsus groups. Twin Block was the most effective appliance, showing higher levels of ALP activity in the first month, third month, and sixth month of sampling compared to the Clear Block and Forsus groups.
